# Efficacy of compressed sodium chloride (CSC) against *E. coli* and *Candida auris* in minutes and methods improvement for testing

**DOI:** 10.1038/s41598-020-79212-2

**Published:** 2021-01-08

**Authors:** Lan N. Truong, Brayden D. Whitlock

**Affiliations:** 1 University of Alberta Health Accelerator, 4560-10230 Jasper Avenue NW, Edmonton, AB T5J 4P6 Canada; 2 Outbreaker Solutions North America, Edmonton, AB Canada

**Keywords:** Infection, Materials for devices, Structural materials, Microbiology, Pathogenesis, Materials science, Civil engineering

## Abstract

Controlling infections has become one of the biggest problems in the world, whether measured in lives lost or money spent. This is worsening as pathogens continue becoming resistant to therapeutics. Antimicrobial surfaces are one strategy being investigated in an attempt to decrease the spread of infections through the most common route of transmission: surfaces, including hands. Regulators have chosen two hours as the time point at which efficacy should be measured. The objectives of this study were to characterize the new antimicrobial surface compressed sodium chloride (CSC) so that its action may be understood at timepoints more relevant to real-time infection control, under two minutes; to develop a sensitive method to test efficacy at short time points; and to investigate antifungal properties for the first time. *E. coli* and *Candida auris* are added to surfaces, and the surfaces are monitored by contact plate, or by washing into collection vats. An improved method of testing antimicrobial efficacy is reported. Antimicrobial CSC achieves at least 99.9% reduction of *E. coli* in the first two minutes of contact, and at least 99% reduction of *C. auris* in one minute.

## Introduction

Infections have become one the biggest problems in the world, and one of humanity’s few existential risks^1^. Antimicrobial resistant (AMR) infections are a particular cause of concern, being projected to cost the world around 2–3.5% of its total GDP, $60–100 trillion USD, and 300 million lives by 2050^2^. Addressing the spread of infections has motivated countless research programs in academia, government and industry.

Pathogens that cause healthcare associated infections (HAIs) place increasing burdens on both patient care and healthcare finances^3,4^. Data from the Centers for Disease Control and Prevention (CDC)’s National Nosocomial Infections Surveillance (NNIS) places HAIs within the top ten causes of death in the U.S.^5,6^, while more recent reports show bloodstream infections (BSIs), a type of HAI, are alone one of the top seven causes of death in North America and Europe, more than influenza and pneumonia combined^7^. The annual direct medical costs of HAIs in U.S. hospitals have been estimated to range between $35.7 and 45 billion^8^. In Europe, HAIs cause 16 million extra patient-days of hospitalization every year^9^. Considering these astronomical costs and the estimation that 70% of infections are preventable^8^, it is clear that HAI prevention should be a public health priority.

In the antimicrobials field, a wide variety of strategies are likely necessary to lower the number and severity of infections. Potential solutions in this space can be broken into two categories: preventative and therapeutic. Ways to clean surfaces and/or make them permanently antimicrobial, part of the prevention effort, is a strategy that has been under investigation for decades with the aim of preventing pathogens from transferring from person to person via the common hand-surface-hand pathway^10^. Currently, most evidence about the routes of transmission for pathogens, including those that can spread through the air via droplets, suggests that the most important route of transmission is via our hands and contaminated surfaces (rather than through aerosolized particles). This can happen either through direct physical contact or through exposure of these surfaces to droplets produced by a cough or sneeze^11^. Only recently has the importance of hand and surface-based transmission been revisited at a global scale as part of infection control advice for the public amid the COVID-19 pandemic^12,13^.

In 2016, a fast-acting, permanent antimicrobial surface made of compressed sodium chloride (CSC) was reported in a pilot study. This antimicrobial CSC was demonstrated to eliminate the AMR bacteria meticillin-resistant *Staphylococcus aureus* (MRSA) more quickly than antimicrobial copper alloys^14^. Sodium chloride was also shown to rapidly inactivate influenza when applied to masks^15^. Given the uncommonly fast action of this surface reported previously, over 20-fold faster than other surfaces, in the present study we characterized this surface further with respect to the speed of action against bacteria. We also report here efficacy against the AMR fungus *Candida auris* and describe a novel method for testing antimicrobial efficacy of surfaces.

## Results

**Figure 1:**
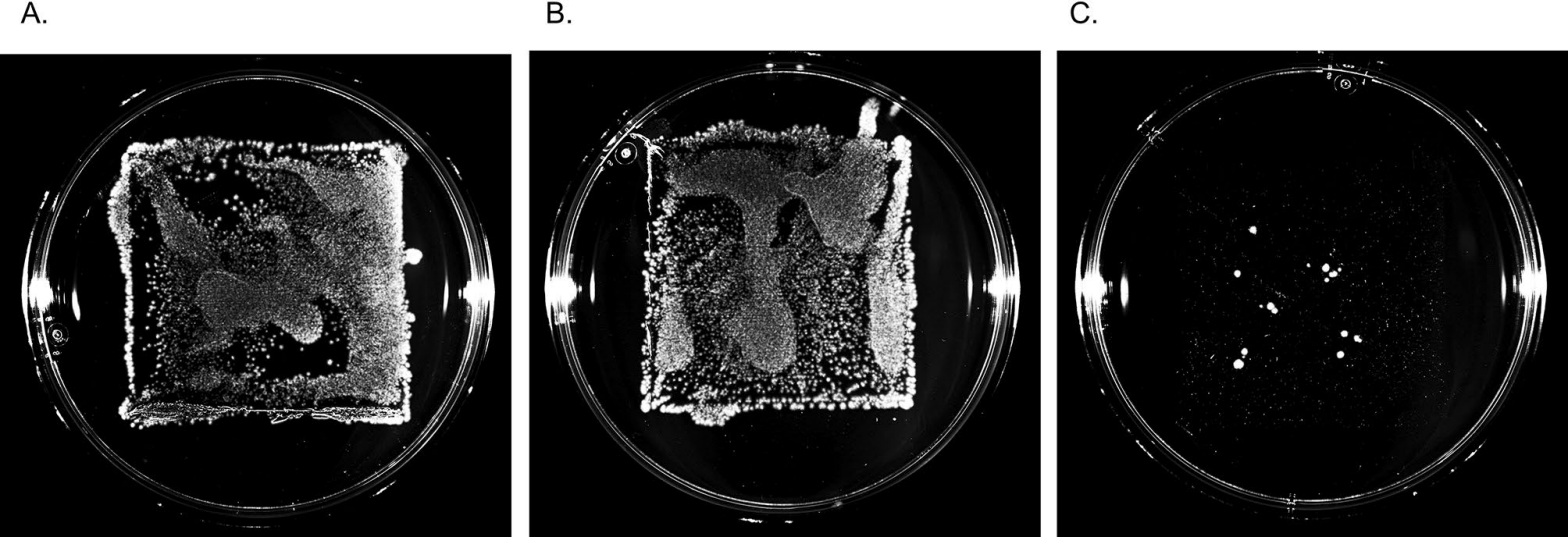
Living bacteria (CFUs) collected from (**A**) stainless steel, (**B**) antimicrobial copper, and (**C**) CSC. The same number of CFUs were added to each surface (~ 150,000), spread, and allowed to incubate at room temperature for 5 s. The surfaces were then stamped with a contact plate for growth and visualization of colonies.

A suspension of *E. coli* containing approx. 150,000 CFUs was added to stainless steel, antimicrobial copper, and antimicrobial CSC surfaces and spread with a sterile spreader. After 5 s, the stainless steel and antimicrobial copper alloy surface transferred an uncountable lawn of bacteria, while the CSC surface transferred very few CFUs (Fig. 1).

Next, stainless steel and antimicrobial CSC surfaces were inoculated with 20 million to 70 million CFUs and allowed to incubate at room temperature for 2 min. Bacteria were then washed off the surface with the wash/scrape collection method, diluted and plated on nutrient agar surfaces for CFU quantification (Table 1). Greater than 99.9% reduction in live bacteria (CFUs) on antimicrobial CSC was observed at two minutes as compared to stainless steel (avg. 99.98%, SD 0.02%). To ensure this wash and scrape method was an effective means of collecting bacteria (i.e. had high sampling efficiency), each surface was stamped with a contact plate after washing to detect, with another method, any bacteria that remained on the surface. In all cases, there was no more than 1 bacteria CFU left behind for every 14,000 added at the beginning, indicating the wash and scrape method was always at least 99.99% efficient.

**Table 1:** Reduction of *E. coli* on antimicrobial CSC vs stainless steel at 2 min measured by the wash and scrape method.

Replicate	CFUs added	CFUs collected		% Recovery (%)		% Reduction (%)
		Stainless steel	CSC	Stainless steel	CSC	CSC vs steel
1	71,000,000	45,250,000	23,000	63.73	0.0324	99.949
2	71,500,000	28,250,000	750	39.51	0.0010	99.997
3	30,000,000	23,000,000	250	76.67	0.0008	99.999
4	23,700,000	6,750,000	1500	28.48	0.0063	99.978
5	61,500,000	23,500,000	7750	38.21	0.0126	99.967
					Average % reduction	99.978
					SD	0.02

The drug resistant fungus *Candida auris* was then investigated in a pilot study on antimicrobial CSC in the same manner that methicillin-resistant *Staphylococcus aureus* was investigated in 2016^14^. Fungus cells were added at either 1:100 dilution (for steel) or undiluted (for CSC), and allowed to contact the surface for one minute. After one minute, the surfaces were sampled by a contact plate and colonies were counted. Out of the 5 experimental replicates, CSC had a reduction vs stainless steel of at least 99% in one minute (avg. 99.24%, SD of 0.16%) (Table 2).

**Table 2:** Reduction of *C. auris* by CSC in one minute measured by the contact plate method.

Replicate	Colony counts		CFUs/ml recovered		% reduction (%)
	Stainless steel diluted 1:100	CSC undiluted	Stainless steel	CSC	CSC vs steel
1	208	165	208,000	1650	99.21
2	212	213	212,000	2130	99.00
3	239	134	239,000	1340	99.44
4	230	168	230,000	1680	99.27
5	320	222	320,000	2220	99.31
			Average % reduction		99.24
			SD		0.16

## Discussion

Here we characterized the speed of action of the new surface, antimicrobial CSC, made of compressed sodium chloride. We also report the first data on antifungal efficacy for CSC.

### Regulatory benchmarks

The speed of action has been under-studied in the field of antimicrobial surfaces, with benchmarks for efficacy set by regulators. For example, the antimicrobial surfaces furthest along in the regulatory process (Environmental Protection Agency in the U.S), antimicrobial copper alloys, make claims of reduction by demonstrating efficacy at two hours^16^. There is some push to move this to one hour^17^. Since the announcement of the first antimicrobial surface registration to make regulated bacterial reduction claims in 2009^18^, much of the work in antimicrobial surfaces has adopted this time point. While reduction times of good antimicrobial surfaces are commonly over an hour, CSC was able to achieve 99.9% reduction of *E. coli* in 2 min^19,20^.

### Modern methods

The methods that have been developed to date are fitting of surfaces that act over the course of hours. However, with antimicrobial CSC, the action takes place over the course of seconds and minutes, necessitating rapid testing methods. Figure 1 is shown as an example of what happens when the surfaces are treated identically, with the same number of CFUs added, using a simple stamp-sampling method. There is no time point at which there are countable colonies on the control surface and the CSC. At any timepoint, either the CSC has countable colonies and the control is vastly overgrown, or the control has countable colonies and the CSC has none remaining. This makes the calculation of % reduction impossible via stamp methods for fast-acting surfaces, if surfaces are treated identically. For slower-acting surfaces, like antimicrobial copper alloys, or for surfaces that do not have any killing effect and merely slow the growth of microbes, like antimicrobial silver coatings, this method is more fitting as there is a small enough difference between CFUs collected from control and treatment surfaces. Thus, stamp/contact plate-based methods allow speed of sampling, but suffer the limitations of how many CFUs can be accurately counted on a single plate. There can be no dilution after the stamp to bring the control CFU values to within a countable range. In order to have countable colonies on all stamps, different concentrations of microbes must be added to the control and CSC surfaces at the beginning, and that dilution is used to correct the counts to CFUs/ml of starting culture. While this method can yield countable CFU numbers for any surface, it requires the surfaces to be treated differently. The effect that seeding density has on the output data cannot be known and cannot be corrected for. It is therefore a good pilot method to determine if the surface should be investigated further, but not sufficient for the full understanding of fast-acting surfaces.

### A new method

The wash and scrape method described herein and used to generate data for Table 1 is an example of a method that cannot be employed as quickly as a stamp method, but which can be much more sensitive. It was developed with thoroughness, not speed, in mind. More microbes can be added to surfaces without risking overgrowth on counting plates, and most importantly, all the surfaces may be treated identically, with no need to add different numbers of microbes to different surfaces. This is because after the microbes are collected from the wash and scrape, they may be diluted by any factor to ensure countable end results, or even monitored by other suspension-based methods that can count living cells specifically. A control surface that would normally have too many CFUs to count via a stamp method is made countable by diluting the collected wash. In this method, we also describe a step to monitor sampling efficiency that ideally would be included in all methods that aim for the highest accuracy and sensitivity. Sampling efficiency, in this case wash efficiency, is a measure of how many bacteria are left behind by the primary sampling method. By determining the proportion of microbes left behind on the surface after washing, a correction can be applied to the data to see if there would be an appreciable change in reduction values had the wash been more efficient. To do this, the number of CFUs collected by the primary sampling method may be inflated to include the CFUs left behind, and the % reduction calculated again to check for any changes. In the present study, the wash was so efficient (> 99.99% in all cases) that correcting for the fact that up to 1 in 14,000 CFUs remain stuck to the surface does not affect the reported data. This step is virtually always omitted in modern antimicrobial surface testing, including regulatory approval protocols. It is instead assumed that the surface is sampled (e.g. washed) at 100% efficiency, or that any difference in sampling efficiency between the control and test surface is negligible.

### Limitations

For future uses of this method and derivatives of it, it should be noted that if a surface is particularly prone to retaining microbes even after the wash and scrape cycles, it is theoretically possible for the reported values of reduction to be skewed by disproportionate microbial retention. This may be particularly relevant to bacteria that form biofilms, especially if the incubation times of the surfaces is long.

The *C. auris* reduction at one minute is to be considered a pilot study, given that it was conducted with a contact plate stamp method. Further research to better understand antimicrobial CSC as a potential antifungal surface could use methods that are sensitive enough to detect greater reduction, such as the wash and scrape method described above. This study has only shown 99% reduction with the pilot stamp method, which is a 2-log reduction. This pathogen was chosen as the representative fungus due to its prominence in healthcare associated infections, but more antifungal research with different species and strains is urgently required.

This study discusses laboratory investigations of an antimicrobial surface, and does not address infectious pathogens passing between people in a clinical setting.

## Conclusion

In this study, we further characterize the antibacterial effect of CSC at short time points and report the first antifungal effect. We describe a new category of antimicrobial surfaces (fast-acting) and methodology to investigate them. Much more research is needed in this field of antimicrobial surfaces in general, with a focus on the understudied fast-acting antimicrobials. Further bacterial, fungal and viral work is ongoing, and we offer to gift surfaces to researchers who wish to replicate our studies, explore new methods, or test CSC efficacy against new microbes.

## Materials and methods

### Materials

Compressed sodium chloride (CSC) was manufactured by Cargill, Incorporated and consists of 97.5–100% sodium chloride compressed into blocks. There are traces of mineral oil from production and natural contaminants in the salt, but there are no active ingredients other than sodium chloride. Sheets of copper alloy C70600 were gifted from Olin Brass (Louisville Kentucky, USA), contain 84.7% copper, and are registered as antimicrobial with the United States Environmental Protection Agency (reg. # 85353-5). Stainless steel controls are NSF food safety grade SAE 304 sheet. The *E. coli* used in this study are ATCC 8739 (Cedarlane, Burlington, Ontario, Canada). The *Candida auris* are UAMH 12148 (MycoBank 508967) from the Gage Research Institute, Toronto, Ontario, Canada. Appropriate contact plates are chosen for the specific pathogen being tested in all cases, following the cell line supplier’s recommendations for growth conditions. Phosphate-buffered saline (PBS) is 137 mM NaCl, 2.7 mM KCl, 11.9 mM phosphates, pH 7.4.

### Contact plate comparison at 5 s

Antimicrobial CSC, sheets of antimicrobial copper alloy, and stainless steel were cut into 5 × 5 cm coupons. All coupons were cleaned with 70% ethanol and exposed to ultraviolet light for ~ 30 min. A ~ 4 h culture of *E. coli* was prepared in sterile nutrient broth (peptone and beef extract) in a shaking incubator (37 °C, 180 rpm). Culture growth was monitored by OD600 reading to yield approximately 700,000,000 colony forming units (CFUs) per ml. Cultures were diluted approximately 1:500 before inoculating surfaces to achieve the desired concentration of 140,000 CFUs/ml, a density optimized for this experiment with the goal of having enough bacteria surviving to count on CSC after 5 s. A 100-ul sample of the diluted culture was applied and spread evenly onto each coupon surface with sterile technique in a biosafety cabinet. The culture and surface were left to incubate at room temperature for 5 s after which an agar (peptone/beef extract) nutrient media contact plate was pressed onto the fixed coupon, ensuring it did not slide. The exact concentration of CFUs in the ~ 4 h *E.coli* culture was determined by diluting the culture to obtain countable CFUs (1:50,000, 1:100,000, 1:500,000), and applying and spreading 100-ul aliquots of each dilution on nutrient media agar plates. The agar plates were incubated at 25 ± 5 °C until colonies were visible on all plates (~ 18 h). Plate images were captured using a Bio-Rad ChemiDoc Imager, using the same settings for all images to make all colonies more clearly visible. This experiment was replicated four times and all the experiments produced similar results.

### Wash and scrape method

A ~ 4 h *E.coli* culture was prepared and monitored via OD600 and via dilution and plating to directly count CFUs/ml, and CSC and stainless steel coupons were prepared as in the contact plate comparison method above. A 100-ul sample of ~ 500,000,000 CFUs/ml culture was deposited and spread evenly on the surface of the coupons using sterile technique in a biosafety cabinet. The culture was left to incubate on the surface at room temperature for 2 min in a biosafety cabinet. At 2 min, coupons were washed with 5 × 5 ml aliquots of sterile phosphate-buffered saline (PBS) projected from a 5-ml pipette. After each wash, the surface was scraped with a sterilized razor blade. The wash solution was collected by gravity and scraping into a sterile basin and the razor blade was deposited in the basin post-wash. Immediately following the fifth razor scrape, the washed coupon surface was pressed onto an agar (peptone/beef extract) nutrient media contact plate, ensuring it did not slide, to monitor the efficiency of the wash method (CFUs left behind after collection vs CFUs added at beginning) and count any CFUs remaining. The contents of the basin were transferred to a 50 ml test tube and vigorously swirled for 1 min. Dilution series were prepared for CSC (undiluted [UD], 1:4, 1:20, 1:100) and stainless steel (UD, 1:4, 1:20, 1:100, 1:1000, 1:10,000) wash solutions. A 100-ul aliquot of each dilution was plated onto an agar (peptone/beef extract) plate. The ~ 4 h *E.coli* culture was diluted as optimized to obtain countable CFUs (1:50,000, 1:100,000, 1:500,000) and 100-ul aliquots were applied and spread onto agar plates. The agar plates were incubated at 25 ± 5 °C until countable colonies were obtained (~ 18 h). The numbers of CFUs on CSC and stainless steel, accounting for the dilution factor, were used to determine the percentage reduction. Values of 5 independent experiments are reported in Table 1.

### *C. auris* reduction by contact plates

This method has been described previously^14^. Briefly, an overnight culture of *Candida auris* was prepared and diluted as optimized to obtain countable numbers of CFUs (undiluted for stainless steel, and 1:100 for CSC). A 100-μl aliquot of the appropriately diluted culture was applied and spread around the entire surface of coupons. The culture was allowed to incubate and dry on the surfaces for 1 min. After the drying time, a tryptic soy agar + lecithin and polysorbate (TSA + LP) contact plate was pressed on to the surface of each sample (CSC) and control (stainless steel), ensuring it did not slide. The plates were incubated at approximately 35 °C until accurate counts could be determined. The numbers of CFUs on surfaces were compared with controls, accounting for the dilution factor, to determine the CFUs/ml recovered, a measure of how many living bacteria were collected per volume of stock culture. Values of 5 independent experiments are reported in Table 2.
